# Increased Risk of Hepatitis B Virus Reactivation in Patients with Resolved Hepatitis B Virus Infection Receiving Combination Immunosuppressive Therapy: The Role of Anti-HBs Status

**DOI:** 10.5152/tjg.2026.26167

**Published:** 2026-06-02

**Authors:** Mustafa Sadeçolak, Tolga Düzenli, İbrahim Durak, Halik Yıldız, Hüseyin Köseoğlu, Bilal Toka, Muhammed Kaya

**Affiliations:** 1 Department of Gastroenterology, Hitit University Faculty of Medicine, Çorum, Türkiye; 2 Department of Gastroenterology, Samsun University Faculty of Medicine, Samsun, Türkiye; 3 Department of Gastroenterology, Medicana International Hospital, Konya, Türkiye

**Keywords:** Antiviral prophylaxis, HBV reactivation, hepatitis B virus, immunosuppressive treatment

## Abstract

**Background/Aims:**

Patients with prior exposure to hepatitis B virus (HBV) (HBsAg [Hepatitis B surface antigen]-negative/anti-HBc [Hepatitis B core] immunoglobulin G (IgG)-positive) who receive immunosuppressive therapy (IST) remain susceptible to HBV reactivation (HBVr). Current clinical guidelines generally estimate HBVr risk according to the immunosuppressive potential of individual agents. However, evidence regarding the cumulative risk associated with the concurrent use of multiple agents within the same risk category remains limited. The current study investigated the effect of frequently used moderate-risk IST combinations on the development of HBVr.

**Materials and Methods:**

A total of 406 HBsAg-negative/anti-HBc IgG-positive patients treated with moderate-risk IST were retrospectively analyzed. Patients were classified according to the treatment regimen into monotherapy (n = 178) and combination therapy (n = 228). HBVr was defined as either HBsAg seroconversion or the detection of HBV DNA during follow-up. The incidence of HBVr was compared between groups.

**Results:**

No significant differences were observed between the groups in baseline demographic and clinical features, including age, sex, and IST-related risk profiles. Among patients receiving combination therapy (58.7% female; mean age 62.8 ± 10.2 years), HBVr occurred in 6 patients, including 4 cases with HBV flare, whereas no reactivation occurred in the monotherapy group (62.3% female; mean age 63.6 ± 10.1 years). All patients who developed HBVr were anti-HBs-negative at baseline. HBVr occurred significantly more frequently among patients receiving combination therapy (*P* < .05).

**Conclusion:**

The use of combination IST and baseline anti-HBs negativity were identified as 2 important factors associated with increased risk of HBVr in HBsAg-negative/anti-HBc IgG-positive patients. In this subgroup, a clinical approach favoring antiviral prophylaxis may be considered. Further prospective studies with larger sample sizes are needed to fully elucidate this association.

**Main Points**

- Patients with resolved HBV infection may experience hepatitis B virus reactivation (HBVr) during immunosuppressive treatment.
- Based on current evidence, HBVr risk is evaluated prior to treatment according to the risk level of the planned immunosuppressive therapy regimen and the patient’s HBV serological profile.
- The findings of the current study suggest that, even within the same risk category, the use of combination immunosuppressive regimens may increase the risk of HBVr.
- Therefore, special attention should be given to antiviral prophylaxis before initiating treatment, particularly in regimens that include combination immunosuppressive agents and in anti-HBsAg-negative patients.

## Introduction

Immunosuppressive therapy (IST) plays a fundamental role in the treatment of many conditions, including solid organ malignancies, hematologic cancers, and rheumatologic diseases.^1^ In individuals with prior exposure to hepatitis B virus (HBV), IST may trigger HBV reactivation (HBVr), a clinically important complication associated with significant morbidity and mortality.^2^ Clinical manifestations of HBVr vary widely, ranging from asymptomatic elevations in liver enzymes to severe hepatitis, liver failure, and death.^3^ Even mild reactivation episodes may necessitate interruption or postponement of IST, thereby delaying treatment of the underlying disease and potentially worsening patient outcomes.^4^

Several international professional societies have issued recommendations for prevention and management of HBVr. Before initiation of immunosuppressive therapy, European, Asian, and American guidelines consistently recommend screening for HBV infection and advocate risk-adapted preventive strategies. Depending on the estimated risk, management may include either prophylactic nucleos(t)ide analog therapy or careful monitoring in selected patients who do not receive antiviral prophylaxis. Preventive antiviral therapy has been shown to markedly reduce the risk of HBVr.^5^^,^^6^ Both HBsAg-positive and HBsAg-negative/anti-HBc immunoglobulin G (IgG)-positive individuals are considered at risk for HBVr.^4^ The risk of HBVr is generally categorized into 3 levels—low (<1%), moderate (1%-10%), and high (>10%)—based on the type of IST and the patient’s serologic status.^7^ Although antiviral prophylaxis is strongly recommended for high-risk regimens, periodic monitoring is generally recommended for low-risk regimens.^8^ Recommendations for the moderate-risk category, however, vary across guidelines, with some advocating prophylaxis and others favoring careful surveillance without antiviral therapy.^8^^,^^9^

In clinical practice, IST for solid organ and hematologic malignancies is frequently administered as combination regimens comprising multiple agents.^10^ In patients with evidence of prior HBV infection (HBsAg-negative/anti-HBc IgG-positive), existing guidelines primarily assess HBVr risk on an individual drug basis. However, combination protocols often involve simultaneous use of multiple low- or moderate-risk agents. Even when all components are within the same risk category, evidence regarding the cumulative risk of HBVr with combination therapy remains limited. Although individual agents may not independently modify management strategies, differences in immunosuppressive mechanisms, potential synergistic effects, and other pharmacodynamic interactions may increase HBVr risk in practice. This uncertainty poses a clinical dilemma and may lead to either unnecessary prophylaxis or unexpected reactivation.

Although HBVr risk assessment is relatively well characterized for monotherapy, accurate stratification in combination regimens remains challenging. Accordingly, the current study aimed to compare HBVr rates between monotherapy and combination IST protocols in HBsAg-negative/anti-HBc IgG-positive patients receiving moderate-risk IST without prophylaxis, and to evaluate the effect of combination treatments on HBVr risk.

## Materials and Methods

### Study Design

This retrospective study included patients treated with IST in the chemotherapy units of Hitit University Faculty of Medicine Training and Research Hospital and Ağrı

Training and Research Hospital between January 2020 and January 2025. Patient data were obtained from electronic medical records and clinical files. Owing to the retrospective study design, written informed consent was not required. Before initiation of IST, all patients underwent HBV screening, including HBsAg and anti-HBc IgG testing. Patients with serological evidence of prior HBV exposure (HBsAg-negative/anti-HBc IgG-positive) who were referred to the departments of gastroenterology or infectious diseases and clinical microbiology were eligible for inclusion. Only patients with confirmed HBsAg-negative and anti-HBc IgG-positive serology were included in the final analysis.

For each patient, the following variables were collected: age, sex, primary diagnosis, IST regimen, HBVr risk classification of the regimen, HBV serology, baseline HBV DNA and serum transaminase levels, consultation notes from gastroenterology or infectious disease specialists, initiation of antiviral prophylaxis, follow-up laboratory parameters, and any evidence of HBVr during follow-up. Patients were categorized as low, moderate, or high risk based on the immunosuppressive agents included in their IST regimens. Patients receiving high- and low-risk regimens and those receiving antiviral prophylaxis within moderate-risk regimens were excluded. Additional exclusion criteria included cirrhosis, prior nucleos(t)ide analog therapy, and prior chemotherapy exposure. In combination regimens, risk classification was determined by the agent with the highest HBVr risk. Risk stratification followed the 2018 guidelines of the American Association for the Study of Liver Diseases (AASLD) guidelines.

The remaining cohort was divided into 2 groups: patients receiving monotherapy and those receiving combination IST regimens. Patients underwent outpatient follow-up at 3-month intervals by gastroenterology or infectious diseases units, including HBV DNA testing and liver function tests. HBVr events occurring during follow-up were recorded. Patients who did not attend follow-up visits or had incomplete laboratory data were excluded from the final analysis. Comparative statistical analyses were performed to evaluate differences in HBVr rates between the 2 groups. The study was conducted in accordance with the Declaration of Helsinki and was approved by the Hitit University Faculty of Medicine Ethics Committee (approval date: February 19, 2026; approval number: 2025-147). No AI was used during the preparation of the manuscript.

### Study Population

Adult patients aged ≥18 years who initiated IST under oncology or hematology clinics were included. Patients with a history of chronic HBV infection, cirrhosis, or HBV antiviral therapy were excluded. Patients with prior IST exposure or antiviral prophylaxis initiated before the current regimen were also excluded. All patients had undetectable baseline HBV DNA before IST initiation. Demographic data, primary diagnoses, serologic results (HBsAg, anti-HBc IgG, and anti-HBs), HBV DNA levels, chemotherapy/IST regimens, and HBVr occurrence during treatment were extracted from the hospital information system and relevant consultation records. Serological status was determined before IST initiation, and prior HBV exposure was defined by anti-HBc IgG positivity. HBVr was defined according to recommendations from American Society of Clinical Oncology, AASLD, and European Association for the Study of the Liver.

### Definitions

HBVr was defined as HBsAg seroconversion and/or reappearance of detectable HBV DNA during the follow-up period. HBV-related hepatitis flare was defined as an alanine aminotransferase (ALT) level ≥3 times the upper limit of normal (ULN) or a ≥3-fold increase from baseline. IST regimens were classified as low, moderate, or high risk according to the 2018 AASLD guidelines.

### Serological and Virological Testing

HBV serological markers, including HBsAg, anti-HBc IgG, anti-HBs, HBeAg, and anti-(Hepatitis B e) HBe, were analyzed using the Roche Modular E-170 electrochemiluminescence immunoassay system (Roche Diagnostics, Basel, Switzerland). Quantification of HBV DNA was performed using the AmpliPrep/COBAS® TaqMan® assay (Roche Diagnostics GmbH, Mannheim, Germany), with a detection range of 15-100 000 000 IU/mL.

### Statistical Analysis

Statistical analyses were performed using SPSS software version 22.0 (IBM SPSS Corp.; Armonk, NY, USA). Continuous variables are presented as mean ± SD and were compared using the Student’s *t*-test. Categorical variables are expressed as percentages and were compared using the chi-square test or Fisher's exact test, as appropriate. Statistical significance was defined as a 2-sided *P* value of less than .05.

## Results

A total of 790 patient records were initially reviewed. Fourteen patients were excluded because of cirrhosis or advanced fibrosis, and 32 were excluded due to receipt of antiviral therapy for HBV. Thirty-three patients were excluded because they received low- or high-risk IST protocols, and 172 patients were excluded because of initiation of antiviral prophylaxis despite being classified as moderate risk. An additional 133 patients were excluded due to missed follow-up visits or incomplete laboratory and clinical data.

**Figure 1.**
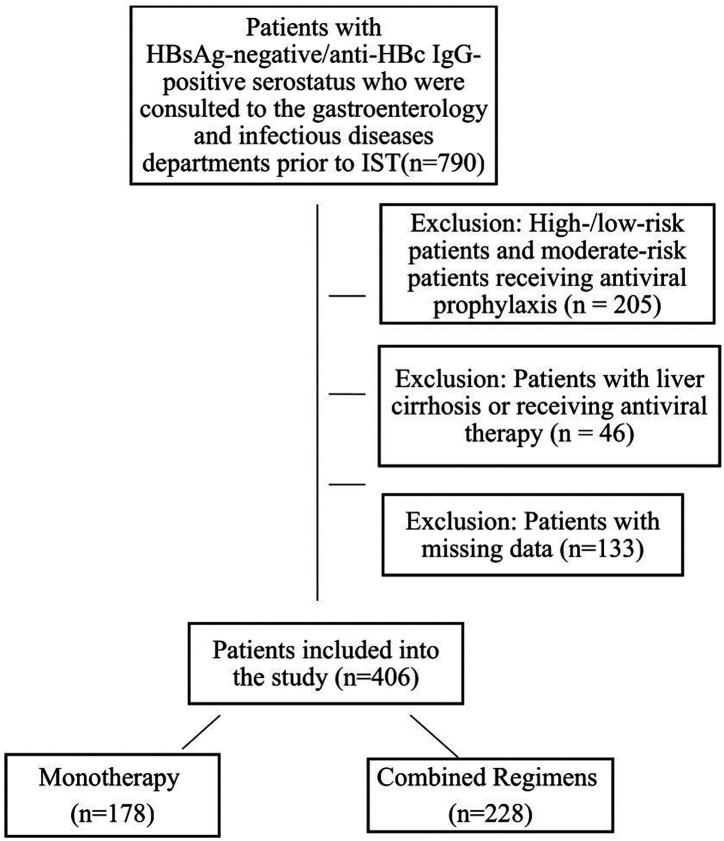
Flowchart of the study.

After these exclusions, a total of 406 patients were included in the study (Figure 1). The mean follow-up duration was 9 months (range: 8-18 months). HBV DNA levels and liver function tests were routinely performed at 3-month intervals in all patients. The mean age of the cohort was 63.2 ± 10.1 years, with 60.3% (n = 245) female and 39.7% (n = 161) male. The majority of patients (97%, n = 394) had solid organ malignancies, whereas 3% (n = 12) had hematological malignancies. Among solid organ tumors, the most common were lung cancer (31%, n=126), colorectal cancer (12.1%, n = 49), and breast cancer (11.3%, n = 46). Hematological malignancies included lymphoma (2.5%, n = 10) and multiple myeloma (0.5%, n = 2). All IST regimens were classified as moderate risk. The most frequently used IST agent overall was the platinum analog cisplatin (34%, n = 138).

Among patients receiving IST monotherapy (n = 178), the mean age was 63.6 ± 10.1 years, and 62.3% were female. All patients had solid organ malignancies and received IST under the supervision of medical oncology. The most common malignancies in this group were lung cancer (26.4%, n = 47), colorectal cancer (14.7%, n = 26), and breast cancer (10.1%, n = 18). The most frequently used immunosuppressive agents in the monotherapy group were cisplatin (22.5%, n = 40), capecitabine (15.7%, n = 28), and gemcitabine (13.5%, n = 24). In the monotherapy group, 75.8% (n = 135) were anti-HBs-positive.

In the combination IST group (n = 228), the mean age was 62.8 ± 10.2 years, and 58.7% were female. Among these patients, 216 (94.7%) received IST for solid organ malignancies and 12 (5.3%) for hematological malignancies. Lung cancer was the most common malignancy (34.6%, n = 79), followed by breast cancer (12.3%, n = 28) and gastric cancer (11.4%, n = 26). The most frequently used combination regimens were cisplatin + etoposide (19.3%, n = 44), paclitaxel + carboplatin (15.8%, n = 36), and cyclophosphamide + doxorubicin (7.5%, n = 17). In the combination therapy group, 73.2% (n = 167) were anti-HBs-positive.

No statistically significant differences were observed between the monotherapy and combination groups with respect to age (*P* = .8) or sex (*P* = .4). All IST regimens were classified as moderate risk, and no differences were detected between groups in risk classification. Anti-HBs positivity rates were similar between groups, with no statistically significant difference (*P* = .55). No cases of HBVr were observed in the monotherapy group. In contrast, HBVr occurred in 2.6% (n = 6) of patients receiving combination IST, which was significantly higher than in the monotherapy group (*P* < .05) (Table 1). All patients who developed HBVr were anti-HBs-negative. Among anti-HBs-negative patients (n = 104), HBVr was observed in 5.7% of patients (n = 6). HBVr occurred significantly more frequently in anti-HBs-negative patients than in anti-HBs-positive patients (*P* < .05).

All patients with HBVr had detectable HBV DNA during follow-up, with a mean HBV DNA titer of 830 893 IU/mL. Of the 6 patients with HBVr, 4 had ALT elevations of ≥3 times the ULN. Antiviral therapy was initiated with tenofovir disoproxil fumarate (n = 3), entecavir (n = 2), or tenofovir alafenamide (n = 1) (Table 2). No HBVr-related mortality was observed; 2 patients with HBV flare required hospitalization and clinical observation. During follow-up of these hospitalized patients, no evidence of acute liver failure was observed. Within 1 month after initiation of antiviral therapy, HBV DNA was undetectable in all patients, whereas elevated serum transaminase levels persisted above ULN in 4 patients with HBV flare. By the second month, serum transaminase levels had normalized in all patients. In the 6 patients who developed HBVr, IST cycles were delayed and standard treatment protocols could not be completed. The IST regimens administered to patients with HBVr included doxorubicin (n = 3), cisplatin (n = 2), cyclophosphamide (n = 2), carboplatin (n = 1), paclitaxel (n = 1), and docetaxel (n = 1).

## Discussion

In the current study, HBVr occurred at a significantly higher rate in moderate-risk IST combination regimens than in monotherapy regimens. Given the high prevalence of HBV exposure in endemic regions and the fact that infection often resolves asymptomatically, a substantial proportion of the general population has an HBsAg-negative and anti-HBc IgG-positive serologic status. Seroprevalence studies have reported isolated anti-HBc IgG positivity in up to 30% of individuals.^11^^-^^13^ In recent years, advances in diagnostic methods, expanded screening programs, and increased life expectancy have contributed to a notable rise in the diagnosis and treatment of both solid organ and hematological malignancies worldwide.^14^ Accordingly, given the prevalence of HBsAg-negative/anti-HBc IgG-positive serology, the potential risk of HBVr during IST is of considerable clinical importance.

HBVr can manifest across a wide spectrum, ranging from asymptomatic transaminase elevations to life-threatening acute liver failure. Even in mild cases, HBVr may necessitate delays in IST and subsequent postponement of primary disease treatment. Current guidelines indicate that HBsAg-negative/anti-HBc IgG-positive patients receiving IST are at risk for HBVr, with risk stratification into low, moderate, and high categories based on the administered agent. Antiviral prophylaxis is recommended for high-risk regimens, whereas low-risk patients may be managed with virological and biochemical monitoring without prophylaxis.^15^ Although recommendations for these 2 extreme risk categories are relatively well defined, management strategies for the moderate-risk group are more heterogeneous, creating uncertainty for clinicians.

Among HBsAg-negative/anti-HBc IgG-positive patients, HBVr associated with rituximab, a high-risk anti-CD20 monoclonal antibody, has been well documented. In a 2024 meta-analysis by Celsa et al,^16^ including 8034 patients across 68 studies, nucleos(t)ide analog prophylaxis was clearly recommended for chemotherapy regimens containing rituximab in HBsAg-negative/anti-HBc IgG-positive patients, whereas antiviral prophylaxis could be withheld in rituximab-free regimens, with prophylaxis considered only in selected patients.^17^ Current guidelines strongly recommend antiviral prophylaxis throughout treatment for regimens containing rituximab.^18^

Many other immunosuppressive agents used for solid organ malignancies fall within the moderate-risk category and are often administered as combination regimens in clinical practice. In centers with high HBVr awareness, there is a tendency to initiate prophylactic antiviral therapy in these patients, which may lead to overtreatment, unnecessary drug-related adverse effects, and increased cost. Conversely, withholding prophylaxis in patients who require it may result in a significant increase in HBVr incidence. Although current guidelines primarily provide recommendations at the monotherapy level for patients at moderate risk, combination IST regimens are frequently used in solid organ malignancies. Careful selection of patients for antiviral prophylaxis is therefore important, as the distinction between those who require prophylaxis and those who can be safely monitored is often subtle. Optimizing patient selection may reduce both HBVr risk and overtreatment. For example, paclitaxel and carboplatin are each categorized as moderate-risk cytotoxic agents; however, no definitive data regarding exist on whether their combination increases HBVr risk in clinical practice. This uncertainty may contribute to variability in clinical decision-making and outcomes.

In the current study, no cases of HBVr were observed in patients receiving moderate-risk monotherapy agents, whereas combination therapy was associated with a statistically significant increase in HBVr incidence. These findings support the recommendation that moderate-risk monotherapy regimens may be safely managed with monitoring alone, whereas combination regimens may warrant prophylactic antiviral therapy. Among the 6 patients who developed HBVr, doxorubicin was included in the IST regimen in 3 cases. The first patient had lymphoma and received doxorubicin combined with bleomycin, vinblastine, and dacarbazine. In the second case, doxorubicin was combined with cyclophosphamide and in the third case with cisplatin. The fourth case received paclitaxel plus carboplatin, the fifth received docetaxel, cisplatin, and fluorouracil, and the sixth received bortezomib, cyclophosphamide, and dexamethasone. In 2021, a study by Tokmak et al^19^ reported no HBVr events among 71 patients receiving doxorubicin + cyclophosphamide and 19 patients receiving paclitaxel + carboplatin regimens. In contrast, the current study observed HBVr in patients receiving similar regimens. HBVr events associated with doxorubicin and cyclophosphamide combinations have also been reported in the literature, including a case in a patient with breast carcinoma receiving doxorubicin + cyclophosphamide + fluorouracil.^20^ Additionally, in 2020, Fidan et al^21^ reported HBVr in 1 patient receiving FOLFIRI (irinotecan + leucovorin + fluorouracil + bevacizumab) in a study of 645 patients with similar HBV serologic profiles.

In the current study, all patients who developed HBVr were anti-HBs-negative. A 2018 prospective study by Tien et al,^22^ including 380 patients, reported that anti-HBs titers ≥100 mIU/mL may confer protection against HBVr. The findings of the current study are consistent with this observation, as no HBVr occurred in anti-HBs-positive patients. Current guidelines also support this observation. Although anti-HBs negativity is recognized as a risk factor for HBVr, European and American guidelines recommend that the decision to initiate antiviral prophylaxis should be guided primarily by the risk level of IST rather than anti-HBs status.^23^^,^^24^

**Figure 2.**
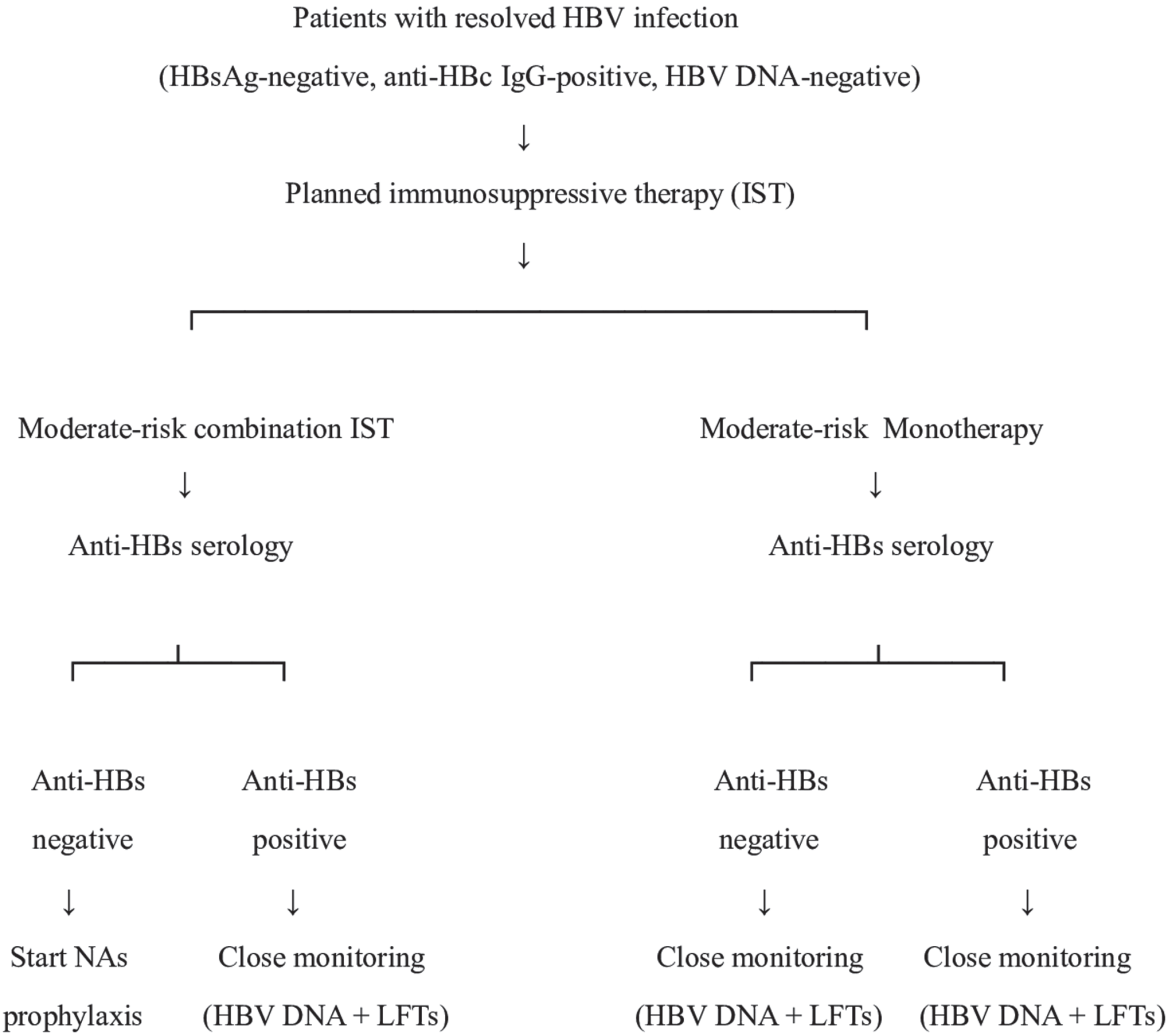
Proposed management algorithm for the prevention of hepatitis B virus reactivation in patients with resolved HBV infection (HBsAg-negative/anti-HBc IgG-positive) undergoing immunosuppressive therapy. HBV, hepatitis B; NAs, nucleot(s)ide analogs; LFTs, liver function tests.

HBVr was significantly more frequent among anti-HBs-negative patients in the current study. These findings highlight anti-HBs status as a potentially important factor in guiding antiviral prophylaxis in patients with resolved HBV infection undergoing combined moderate-risk IST. Notably, HBVr occurred exclusively in patients receiving combination regimens, highlighting the increased immunosuppressive burden associated with these treatments. In this context, anti-HBs-negative patients appear to be at higher risk and may benefit from routine antiviral prophylaxis. Conversely, anti-HBs-positive patients may be candidates for a preemptive monitoring strategy, although this approach requires strict virological surveillance, as protective immunity is not absolute. These data support a more individualized approach, in which anti-HBs status is incorporated into prophylaxis decision-making for patients receiving combination IST. Based on the results of the current study, initiating nucleos(t)ide analog prophylaxis may be considered in anti-HBs-negative patients receiving combination IST regimens. In contrast, patients receiving monotherapy, particularly those who are anti-HBs-positive, may be safely managed with monitoring alone without antiviral prophylaxis (Figure 2).

Another important consideration in assessing HBVr risk is the type of malignancy requiring IST. In the current study, 2 of the 6 HBVr cases occurred in patients with hematological malignancies. In a 2018 meta-analysis conducted by Cholongitas et al,^25^ including 3640 HBsAg-negative/anti-HBc IgG-positive patients, the pooled HBVr rates were reported as 10.6% in patients with hematological malignancies and 3.6% in those with nonhematological malignancies. However, it has been suggested that this difference may be more closely related to the content and intensity of the administered IST rather than the type of malignancy itself. As noted above, high-risk IST regimens significantly increase the likelihood of HBVr, whereas the distinction between hematological and nonhematological malignancies has not consistently demonstrated a statistically significant impact on HBVr risk. Therefore, further randomized controlled studies are warranted to clarify the independent effect of malignancy type on HBVr.

The current study focuses on patients with resolved HBV infection and provides novel insight by comparing monotherapy and combination IST regimens, supported by a relatively large sample size and standardized follow-up. In addition, anti-HBs negativity was associated with an increased risk of HBVr, contributing to risk stratification. As anti-HBs status may represent a confounding factor in HBVr risk, randomized controlled studies are needed to more precisely assess the independent effect of combination IST. However, the retrospective design, limited number of centers, and the relatively small number of HBVr events may limit the generalizability and statistical power of the findings, and the lack of randomized comparisons precludes definitive conclusions.

In conclusion, the findings of the current study suggest that the cumulative immunosuppressive effect of combination regimens may increase the risk of HBVr. Based on these findings, nucleos(t)ide analog prophylaxis should be considered in patients with resolved HBV who are anti-HBs-negative and are scheduled to receive moderate-risk combination IST. In contrast, close monitoring may be an appropriate strategy for anti-HBs-positive patients receiving monotherapy.

## Figures and Tables

**Table 1.** Clinical Characteristics of the Monotherapy and Combined Regimen Groups

		Monotherapy [n (%)]	Combined regimens [n (%)]	*P*
Total [n (%)]		178 (43.8)	228 (56.1)	
Age (mean ± SD)		63.6 ± 10.1	62.8 ± 10.2	.8
Gender (female/male)		111/67	134/94	.42
HBVr risk of the protocol (moderate/high)		178/–	228/–	.95
Underlying diseases	Total	178	228	
	Lung	47	79	
	Colorectal	26	23	
	Breast	18	28	
	Hematologic malignancy	–	10	
	Others	87	88	
Anti-Hbs (Antibody to Hepatitis B surface antigen) seropositivity		135 (75.8)	167 (73.2)	.55
HBVr		–	6	<.05

HBV, hepatitis B virus; HBVr, HBV reactivation.

**Table 2.** Clinical Outcomes of Patients Developing HBVr

Age (years)	1	2	3	4	5	6
	77	74	48	77	67	55
Sex (F/M)	M	F	M	F	M	M
Diagnosis	Lymphoma	Multiple myeloma	Lung cancer	Breast cancer	Osteosarcoma	Gastric cancer
IST regimen	Doxorubicin Bleomycin Vinblastine Dacarbazine	Bortezomib Cyclophosphamide Dexamethasone	Paclitaxel Carboplatin	Cyclophosphamide Doxorubicin	Doxorubicin Cisplatin	Docetaxel Cisplatin Fluorouracil
HBV DNA level during HBVr (IU/mL)	2 870 000	18 400	659	1 688 000	75 300	333 000
HbsAg seroconversion during HBVr	+	+	+	+	+	+
ALT level (U/L)	286	95	478	42	24	117
Treatment	TDF	ETV	TDF	TAF	ETV	TDF

ALT, alanine aminotransferase; ETV, entecavir; F, female; HBV, hepatitis B virus; HBVr, HBV reactivation; IST, immunosuppressive treatment; M, male; TAF, tenofovir alafenamide; TDF, tenofovir disoproxil fumarate.

## Data Availability

The data that support the findings of this study are available on request from the corresponding author.
